# Supplementary material for: Mapping Human Laryngeal Motor Cortex during Vocalization
Source: Cereb Cortex. Author manuscript; Available in PMC 2021 Apr 26. (PMC7610685; doi:10.1093/cercor/bhaa182)
Supplement: Supplementary Data — Supplementary Material. Supplementary material can be found at Cerebral Cortex online. [file EMS122731-supplement-Supplementary_Data.docx]

# **Supplementary Material For:**

# **Mapping human laryngeal motor cortex during vocalization**

Nicole Eichert^1^, Daniel Papp^1^, Rogier B. Mars^1,2^ & Kate E. Watkins^3^

^1^ Wellcome Centre for Integrative Neuroimaging, Centre for Functional MRI of the Brain (FMRIB), Nuffield Department of Clinical Neurosciences, John Radcliffe Hospital, University of Oxford, Oxford, United Kingdom

^2^ Donders Institute for Brain, Cognition and Behaviour, Radboud University Nijmegen, Nijmegen, The Netherlands

^3^ Wellcome Centre for Integrative Neuroimaging, Department of Experimental Psychology, University of Oxford, Oxford, United Kingdom

Correspondence:

Nicole Eichert

Wellcome Centre for Integrative Neuroimaging

Nuffield Department of Clinical Neurosciences

John Radcliffe Hospital, University of Oxford

Oxford OX3 9DU

United Kingdom

Direct Line: +44 (0) 1865 271363

E-mail: [nicole.eichert@psy.ox.ac.uk](mailto:nicole.eichert@psy.ox.ac.uk)

**Surface count maps of all individual task contrasts**


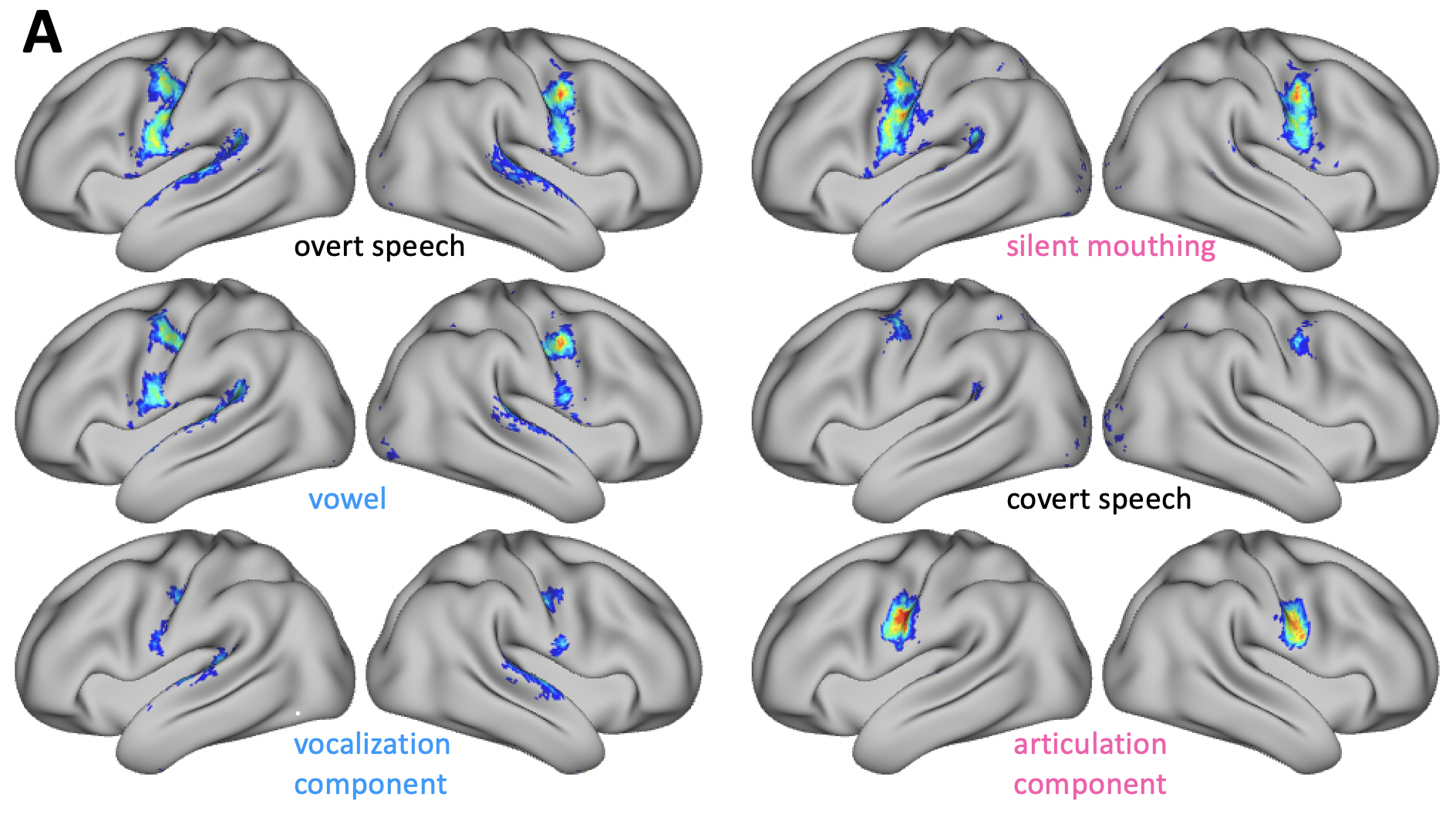


**
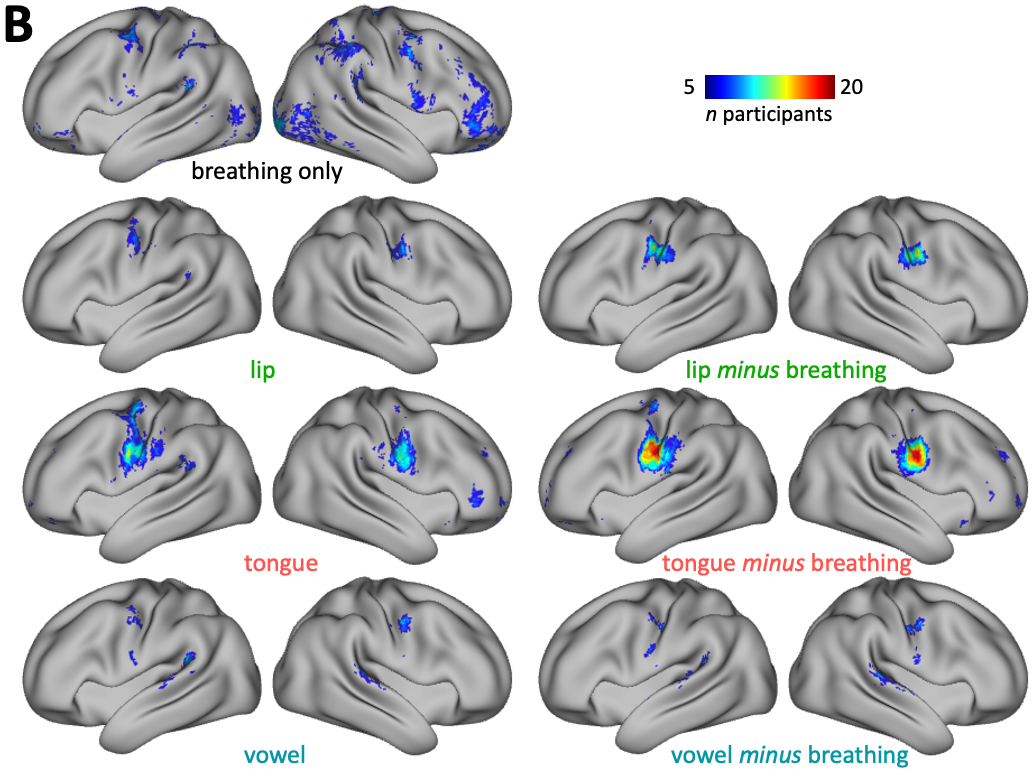
**

Figure S1 – Related to Figure 2 and Figure 3. Surface count maps of all individual task contrasts. **A**: Syllable production task. Upper two rows: Four task conditions - overt speech, silent mouthing (articulation only), vowel production (vocalization only), covert speech (thinking) when compared to the resting baseline with normal breathing. Third row: Two orthogonal main contrasts for the articulation and vocalization component (these are also shown in Figure 2B). **B**: Basic localizer task. Left panel: Task conditions compared to the resting baseline with normal breathing. The ‘breathing only’ condition is a contrast between the breathing condition (where breathing was instructed) and the resting baseline with normal breathing. A lower threshold of *z* = 1.96 was used to threshold individual *z*-statistical images for this contrast due to generally lower activation. Right panel: Movement conditions compared to the breathing condition with explicit breathing instruction (these are also shown in Figure 3B).

**Task activations in supplementary motor area**

The two main contrasts for articulation and vocalization - at a lower voxel-wise threshold of *z* > 2 - revealed activity bilaterally in SMA (Figure S2). A somatotopic arrangement was observed with vocalization activating a more anterior and more dorsal part of cortex. The representations of the effectors during the basic localizer task also show a clear somatotopy: In dorsal-to-ventral direction we first find the representation of the larynx, which is also most anterior, then of the lip and then of the tongue. Only one single representation of the larynx was found in SMA in both the main contrast for vocalization and in the vowel condition.

The location of the representation of the speech effectors is in line with previous accounts in the literature (Picard and Strick 1996). It has been suggested that the vertical line crossing the anterior commissure, the VAC, is a landmark for a division of SMA proper and pre-SMA (Picard and Strick 1996; Rizzolatti et al. 1996). Our results thus suggest that lip and tongue representations are located just posterior to VAC in an anterior region of SMA proper. Laryngeal activity during vocalization, however, activates a part of cortex anterior to VAC, presumably in pre-SMA.

**Task activations in cerebellum**

Movement of the articulators and laryngeal activity also evoke activation of the cerebellum in a somatotopic fashion, which is mirroring the order observed for motor cortex (Figure S2). Most ventrally, a representation of the larynx is observed, which activates during vowel production and vocalization. This is followed dorsally by a representation of the lips and the tongue and then by a second representation of the larynx.

According to a probabilistic atlas of the human cerebellum (Diedrichsen et al. 2009), all the activations observed are located in the anterior cerebellar lobule VI. This finding is consistent with previous neuroimaging studies that found activation of the anterior-superior aspect of cerebellum during speech movements (Petersen et al. 1989; Fiez and Raichle 1997). A previous resting-state functional connectivity study demonstrated that cerebellar representations mirror the topography in cerebral motor cortex (Buckner et al. 2011). It is therefore presumed that the ventral-most representation of the larynx in cerebellum is related to the dorsal larynx representation in motor cortex and vice versa.


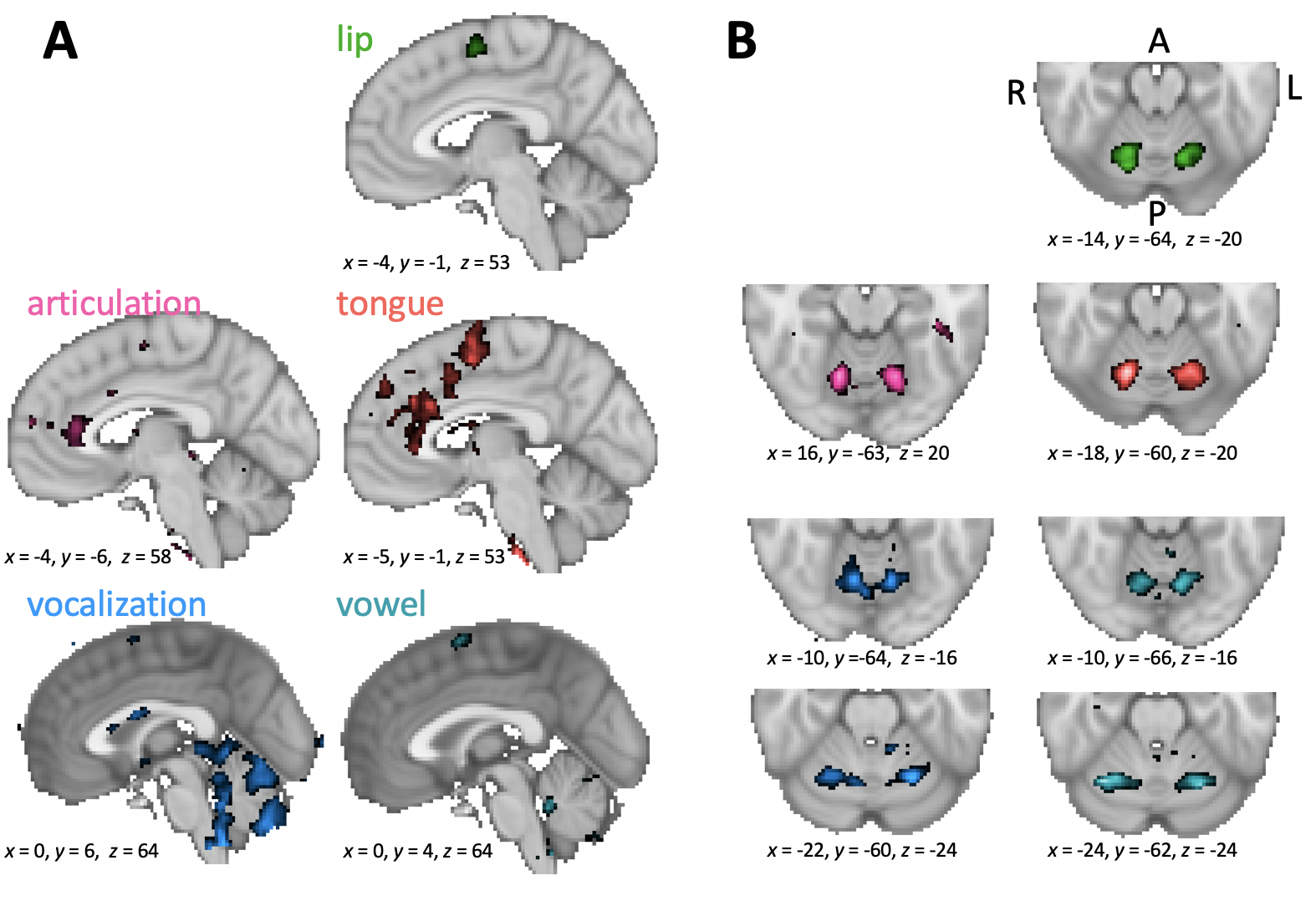


Figure S2 – Related to Figure 2 and Figure 3. Whole-brain group activation maps showing areas activated during syllable production task (left panels) and basic localizer task (right panels) (vowel-wise threshold *z* > 3.5, *n* = 20). Coordinates are given for the voxel of maximal activation in the left hemisphere. **A**: Activity on the medial brain surface centered on the voxel of maximal activity in the left supplementary motor area (SMA) (note that for the main contrast for vocalization the threshold was lowered to *z* > 2). **B**: Cerebellar activity in the same task contrasts and shown in the same colors as in **A**. For the main contrast for vocalization and for the vowel production contrast, two separate activations are shown in different slices.

**Regions-of-interest to derive maxima during task activation**

### An example of the volumetric ROIs in an individual is shown in Figure S3. The central sulcus ROI used for the hand, lip and tongue was defined using FreeSurfer’s automatic volumetric labelling based on the Destrieux Atlas.

For the larynx, we identified two activation maxima in separate ROIs: One for the dorsal and one for the ventral larynx representation. The dorsal larynx ROI was a portion of the same central sulcus ROI used above from *z*-coordinates in MNI space of 50 - 30. The limits were determined empirically, so that ROI did not capture the ventral larynx representation or an unrelated supra-dorsal activation in the trunk area, which was observed in some individuals (Foerster 1931).

The ventral larynx representation lay outside the central sulcus and was located ventrally in the subcentral part of cortex. Due to the high intra-individual morphological variability in this region (Eichert et al. 2020), the ventral larynx ROI was derived manually based on individual anatomy in surface space. A liberal surface ROI was drawn on each individual’s midthickness surface covering the ventral part of the central sulcus and adjacent gyri (Figure S3A). Anteriorly, the ROI was delineated by the inferior portion of the precentral sulcus and posteriorly the ROI spanned the postcentral gyrus. If present, the lateral portion of the ascending sulcus in the subcentral gyrus was included within the ROI. The dorsal limit of the ROI was defined by a horizontal plane across the gyrus at the level of the usual location of the posterior ramus of the inferior precentral sulcus. The ventral larynx surface ROI was converted into a volumetric ROI covering the underlying cortical ribbon using wb_command. We checked that the ventral larynx ROI did not overlap with subjacent auditory cortex in the temporal lobe or inferior frontal cortex.

In some subjects, the main contrast for vocalization in the syllable production task had additional activity related to articulation of the tongue. To remove this, we transformed the coordinates for each individual’s maximal voxel from the tongue contrast (from the basic localizer task) to the functional space of the syllable production task (task 1) using rigid-body transformation and then derived a spherical ROI (7 voxels diameter) around it. This sphere was used to mask the *z*-statistic image of the main contrast for vocalization prior to localizing the maxima for laryngeal activity in the dorsal and ventral ROIs described above.


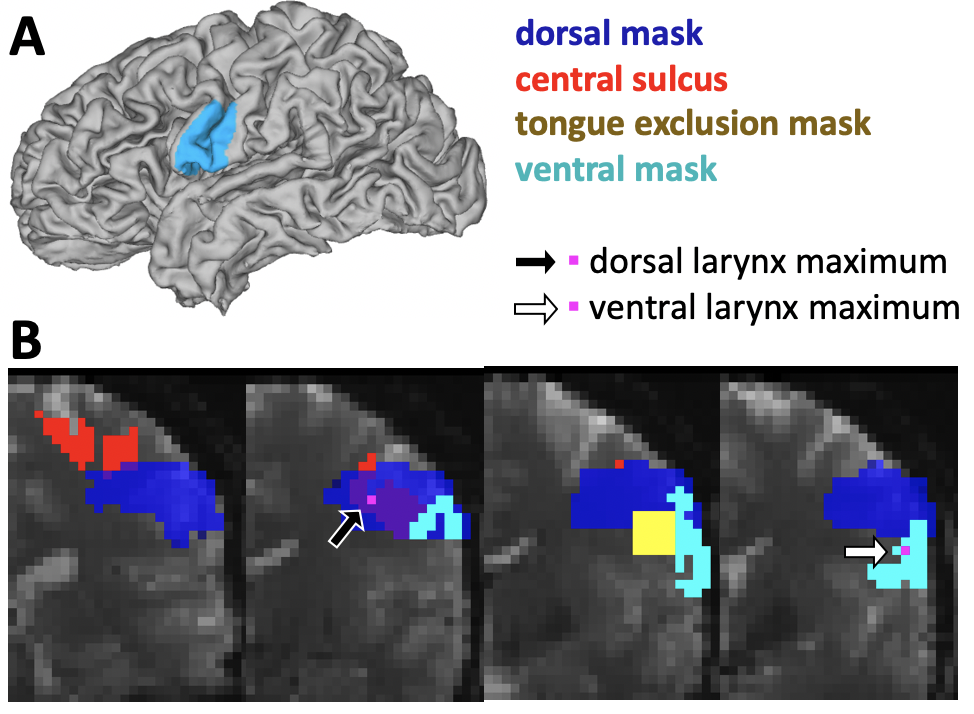


Figure S3 – Related to Methods. **A**: Example of a manually-drawn surface ROI for the ventral larynx representation. **B**: Volumetric ROIs overlaid onto an individual’s functional scan. The central sulcus ROI (red) was used to derive the voxel of maximal activation for the hand, lip and tongue. The intersection of the central sulcus ROI and a dorsal mask (dark blue) was used to derive the voxel of maximal activation for the dorsal larynx representation (black arrow, pink voxel). The ventral mask was projected from surface to volume space (bright blue) to derive the voxel of maximal activation in the ventral larynx representation (white arrow, pink voxel). A spherical ROI around the voxel of maximal activation in the tongue contrast was masked out from the dorsal and ventral mask (yellow).

**Group-level task activation maxima**

Table S1: Group-level task activation maxima. Reported are MNI coordinates of maxima in somatomotor cortex for the main contrasts reported in the manuscript (LH, RH: left and right hemisphere). For the main contrast for vocalization and vowel production, a dorsal and a ventral maxima are reported separately.

| task | main contrast |  | LH |  |  | RH |  |
| --- | --- | --- | --- | --- | --- | --- | --- |
|  |  | *x* | *y* | *z* | *x* | *y* | *z* |
| hand localizer | hand | -38 | -21 | 57 |  |  |  |
| syllable production | articulation | -50 | -12 | 32 | 54 | -2 | 32 |
|  | vocalization (dorsal) | -40 | -16 | 36 | 43 | -13 | 38 |
|  | vocalization (ventral) | -58 | -2 | 18 | 60 | -2 | 16 |
| basic localizer | lip | -48 | -10 | 40 | 50 | -8 | 36 |
|  | tongue | -52 | -11 | 28 | 56 | -4 | 30 |
|  | vowel (dorsal) | -42 | -16 | 40 | 50 | -6 | 40 |
|  | vowel (ventral) | -58 | -2 | 20 | 58 | -4 | 18 |

## **Supplementary References:**

Buckner RL, Krienen FM, Castellanos A, Diaz JC, Yeo BTT, Thomas Yeo BT. 2011. The organization of the human cerebellum estimated by intrinsic functional connectivity. Journal of Neurophysiology. 106:2322–2345.

Diedrichsen J, Balsters JH, Flavell J, Cussans E, Ramnani N. 2009. A probabilistic MR atlas of the human cerebellum. NeuroImage. 46:39–46.

Eichert N, Watkins KE, Mars RB, Petrides M. 2020. Morphological and functional variability in central and subcentral motor cortex of the human brain. bioRxiv. 2020.03.17.995035.

Fiez JA, Raichle ME. 1997. Linguistic Processing. In: International Review of Neurobiology. p. 233–254.

Foerster O. 1931. THE CEREBRAL CORTEX IN MAN. Lancet. 309–312.

Petersen SE, Fox PT, Posner MI, Mintun M, Raichle ME. 1989. Positron emission tomographic studies of the processing of single words. Journal of Cognitive Neuroscience. 1:153–170.

Picard N, Strick PL. 1996. Motor areas of the medial wall: A review of their location and functional activation, Cerebral Cortex. Narnia.

Rizzolatti G, Luppino G, Matelli M. 1996. The classic supplementary motor area is formed by two independent areas. Advances in neurology. 70:45–56.
